# Pathology and Therapeutics

**Published:** 1859-05

**Authors:** 


### PATHOLOGY AND THERAPEUTICS.

### V.—*Observations on Chorea Magna.* By PROF. SKODA.

Chorea magna differs from the so-called chorea minor only in its paroxysmal character, while the latter consists of continued clonic spasms. It is indifferent what kind of movements take place; they may vary very much, and either consist in springing, turning, or weltering, rolling, etc. The movements are commonly very violent, but in spite of continuing for one hour or longer, are not followed by exhaustion or pain. The disease is distinguished from paralysis agitans by the trembling movements characteristic of the latter, while in chorea the limbs are moved in a quite normal manner. The cause of chorea magna is still unknown, but so much may be stated in reference to it, that the changes of the brain or spinal marrow in this disease, as in chorea minor, can be of but insignificant character, while in paralysis agitans the disease of these structures is by far more advanced. From the temporary appearance of chorea magna, it seems probable that not so much organic disease of the nervous centres, particularly of the brain, is present, the disturbances of the latter alone being sufficient to explain all the symptoms,—as merely functional alteration, particularly of the organs presiding over motion, though sensation is also frequently affected. This anomaly of function is probably the reason that chorea magna is cured more easily than chorea minor, which runs its course generally in a definite length of time. Relapse is equally common in both diseases; great caution is, therefore, necessary after the cessation of the malady.

The best remedy is *quinia*, small doses of which are sometimes sufficient to effect a cure, but often the dose must be raised to thirty-six grains. In order to avoid a relapse, these large doses must be several times repeated, as in intermittent fever. They are generally borne without bad consequences.—(*Allgem. Wien. med. Ztg.* 36, 1858, and *Schmidt's Jahrbücher*, 1859, I.)

VI.—*Observations on the Etiology and Treatment of Hemicrania.* By DR. MERZ, of Böhrenbach.

The author has made the observation, important in a practical point of view, that hemicrania is curable by compression of the carotid artery of the affected side; in order to make the cure permanent, the compression must be continued for a length of time; a temporary use of the measure will afford only transitory relief. The shortest duration of the compression is fifteen minutes. The cause of this influence the author assumes to be the diminished supply of arterial blood to the vessels of the base of the brain; in three fatal cases he found these vessels in a state of aneurismal enlargement. Although this enlargement remains without influence upon the cerebral functions as long as the pressure of the blood is normal, it occasions a considerably increased flow of blood to the brain, with its evil consequences, whenever the pressure is augmented. The author points out that analogous changes take place in epilepsy, in which disease Schroeder van der Kolk has observed an increased afflux of blood to the medulla oblongata. Dr. Merz is, however, by no means inclined to recognize this as the only cause of hemicrania.

The pressure is exercised either with the thumb, or with a compressor made of a powerful spring, and shaped like a hernia-truss. The instrument is adjusted upon the muscles of the neck, its pad resting upon the carotid at the point where the vessel emerges from under the sterno-cleido-mastoideus. Dr. Merz reports twenty-four cases, which speak very favorably for his method.—(*Aerztl. Mitthlg. a. Baden*, xii. 15, 1858.)

VII.—*Water-Glass, a Partial Substitute for Collodium.* By DR. KUCHENMEISTER.

*Preparation of water-glass*, (Lehmann's Taschenbuch d. theor. Chem.) If ten parts of potassa are melted together with fifteen parts of powdered quartz, and one part of charcoal, a blackish-gray glass is obtained which is soluble in five parts of water; on evaporating this solution, an opalescent, semi-fluid mass is formed, which has an alkaline taste and reaction, and does not absorb carbonic acid from the air; by slow evaporation it is formed into a glassy mass of conchoidal fracture, unchangeable at the atmosphere; it is this water-glass= $3KO,8SiO_3$. A similar substance is obtained with soda.

The author has used this substance as an external application in cases of bee-sting, with excellent result, and has found it to diminish the pain and swelling very promptly; he explains its efficacy partly by the circumstance that the alkaline water-glass neutralizes the acid of the bee's poison (formic acid,) partly by the fact that on evaporating slowly on the skin—similar to collodium, but somewhat slower—it forms a smooth and even coating, which protects the wound from the entrance of air, dust, and other foreign substances. Water-glass may thus be usefully employed:—

1. *In cases of bites and stings of such animals which introduce an acid poison into the wound*, viz., bees, bumble-bees, wasps, hornets, gnats, mosquitoes, bugs, toads, perhaps also snakes. But the author recommends the remedy particularly in cases in which ticks, sand-bugs, lepti autumnales, and crab-lice have bitten themselves into the skin, as the removal of the parasite is much facilitated by the application, the coat formed by the water-glass suffocating the animal by obstructing the tracheal openings which project from its body.

2. The application of water-glass proved also very efficacious in a case of *erysipelatous inflammation* of the hand in a child; in erysipelas ambulans of the face, the author has not been so successful with the remedy. Whether it could be used with advantage in mastitis, skin diseases of different nature, particularly those of the humid kind, or in tetter with an acid reaction, remains to be ascertained; in herpes circinatus it is very useful.

3. Water-glass is one of the best means *to cleanse the skin* from tar, varnish, residues of plaster, etc.; it may perhaps be also usefully employed to clean the scalp in diseases of the hair.

*It is inferior to collodium in respect* to tenacity, and is, therefore, less applicable in gaping wounds; in cases in which a coating is required to be durable if immersed in water, it cannot be used at all. In order to avoid its crumbling off, the coating ought to be renewed from time to time. *It is again preferable to collodium* on account of the greater readiness with which it can be removed.—(*Memorab. a. d. Prax.* iii. 8, and *Schmidt's Jahrbücher*, 1859, I.)

### VIII.—*Ethereal Oil of the Horse-Chestnut as a Topical Application in Gout and Rheumatism.* By MM. GENEVOIX and CH. MASSON.

On extracting powdered horse-chestnuts with sulphuric ether, and on evaporating the latter, an oil is obtained (about one drachm from ten pounds and a half of horse-chestnuts) which has proved a useful application in gout and rheumatism. The oil is painted several times in quick succession upon the suffering part, or around it, if it is too sensitive; the painted part is then covered with blotting-paper, over which cotten or flannel, and oiled silk is applied. The dressing may be renewed several times daily, according to necessity. During the first half hour the pain is often, though not always, augmented, but decreases afterwards quickly, and ceases entirely.—(*Bull. de Thér.* lv. p. 217, September, 1858.)

### IX.—*Ointment for Sore Nipples.* By DR. VAN HOLSBECK.

Dr. van Holsbeck recommends the following mixture, which he has used with much success in the treatment of sore nipples: Oleum cadinum, 3½ parts; Ol.

amygdal. dulc., 3 parts; and Glycerin, 3 parts. If the fissures in the nipple are very deep or large, the quantity of the Ol. cadinum must be increased, and the mixture must be applied with a camel's-hair pencil each time after the child has taken the breast. The odor is not unpleasant to the infant, and the application does not occasion pain, but only a gentle heat; the fissures heal within a few days.—(*Preuss. Vereinsztg.*, July 21, 1858.)

X.—*On the Diuretic Properties of Coffee.* (Allgemeine medizin. Centralztg. November, 1858.)

As early as the year 1725, Dr. Th. Zwinger, a Swiss physician, recommended coffee as a remedy in dropsy. More recent investigations have proved that by the use of coffee the amount of water in the urine is increased, while urea, phosphoric acid, and other substances are rather diminished, and consequently remain in the body, a circumstance which may serve to explain the nutritious properties of coffee. As a diuretic, coffee ought not to be given with milk, this mixture producing in many persons flatulence and diarrhœa, but as pure decoction which, as is well known, exercises a tonic influence upon the intestinal canal. As a special remedy in dropsy, coffee is hardly known at the present time.

XI.—*On the Topical Use of Laudanum in Affections of the Eyes.* By Prof. Nasca, of Naples.

Prof. Nasca is of the opinion that the local application of laudanum in diseases of the eyes is at present too much neglected. Acute inflammation of the conjunctiva contra-indicates the use of this remedy; as soon, however, as the acute state has been removed by the antiphlogistic treatment, and the inflammation assumes the chronic character, laudanum is very efficient. For about a week one drop of the preparation is dropped into the eye once every day, and the quantity is gradually increased to two, three, and four drops daily. The use of emollient lotions and fomentations is to be entirely avoided, but light wine, with a small addition of tincture of myrrh, will be found a very serviceable application.

The author derived, also, great advantage from the external use of laudanum in the case of old persons whose faculty of vision was growing feeble, and seemed to require the aid of convex glasses. The patients were ordered to rub laudanum on the forehead and eyelids every night, and not to remove the application until the next morning; after continuing this treatment for about thirty to forty days, the patients generally found their sight improved in a remarkable degree. This method can be, of course, only successful in cases in which there exists no divided material alteration in the nervous apparatus of the eye.—(*Gaz. Médicale de Lyon*, November, 1858.)

